# Pyometra Puzzler: Unraveling Senile Endometritis in an Unconventional Presentation

**DOI:** 10.7759/cureus.61394

**Published:** 2024-05-30

**Authors:** Lucky Srivani Reddy, Manjusha Agrawal, Arpita Jaiswal, Kamlesh Chaudhari, Apoorva Dave

**Affiliations:** 1 Obstetrics and Gynaecology, Jawaharlal Nehru Medical College, Datta Meghe Institute of Higher Education and Research, Wardha, IND

**Keywords:** post-menopause, uterine rupture, pyometra, geriatric, intrauterine infection

## Abstract

Pyometra is a very uncommon condition in postmenopausal women that rarely improves with standard antibiotic treatments. It is usually overlooked as the patient presents with vague symptoms. Our case presented a postmenopausal woman with sepsis due to a huge pyometra. Swabs for sensitivity, tubercular gene testing, and basic blood workup were done, and the patient was started on intravenous antibiotic therapy. Pyometra drainage could not be done due to thin, friable uterine walls. When the patient had improved, a clinically total abdominal hysterectomy was done after ruling out malignant causes. Delay in the diagnosis of this condition may lead to perforation, which may, in turn, cause peritonitis, which may gravely affect the patient.

## Introduction

Pyometra is the accumulation of purulent material in the uterine cavity due to the blockage of the natural drainage of the uterus; it is usually seen among 0.01-0.5% of gynaecological patients [1,2]. The majority of pyometra cases occur among older, postmenopausal women who have had recent intrauterine surgery or who have concomitant conditions, including high blood pressure, rheumatoid arthritis, osteoarthritis, or poor glucose tolerance [3]. The use of an intrauterine device (IUD), radiation cervicitis, atrophic cervicitis with ageing, and benign or malignant gynaecologic tumours are the primary contributors to this illness [4-6]. Lower abdominal pain, postmenopausal bleeding, and purulent vaginal discharge are the classical triage of symptoms among pyometra patients [4]. The most common side effects of pyometra are bacteremia, sepsis, and spontaneous uterine rupture resulting in extensive peritonitis [1]. Postmenopausal cervical stenosis can make pyometra draining and hysteroscopic examination challenging which may even lead to perforation of the uterus further complicating the case [7]. When pyometra is suspected, the best imaging modalities for identifying this infection are an MRI, CT, and ultrasound [8]. A patient presenting with pyometra of bacterial origin due to senile endometritis with cervical stenosis is being discussed.

## Case presentation

A 60-year-old lady presented to the emergency department with complaints of vaginal discharge for the past 1.5 years, which was purulent for the past one month. The purulent discharge has been associated with abdominal fullness, sharp pain and itching for the past one month. She had high-grade fever, chills and rigours for the past two weeks. Her examination revealed a fever of 100 degrees Fahrenheit, a steady pulse of 108 beats per minute, a respiratory rate of 24 breaths per minute, and a blood pressure of 110/70 mm Hg. The cardiovascular, pulmonary and central nervous system examination findings were normal. An initial blood work-up was sent, which revealed haemoglobin of 11.5 gm% and leucocyte counts of 18,000/cum (Table 1). On abdominal examination, it was soft and non-tender. Per speculum examination revealed a pale vagina, small atrophic cervical os, and pustular discharge through the os. A swab of the discharge was taken. Per vaginal examination small uterus was atrophied and retroverted, bilateral fornices were free, and no adnexal masses were palpable.

**Table 1:** Lab results from the blood investigations were obtained.

Investigations	Lab values of the patient	Normal range in adult female
Hemoglobin (gm%)	11.5	12-15
Total leucocyte count (/cumm)	18,000	4,000-10,000
Cancer antigen 19.9 (CA 19.9) (U/mL)	2	<37
Carcinoembryonic antigen (CEA) (ng/mL)	0.1	<3
Cancer antigen 125 (CA 125) (U/mL)	8	<35
Lactate dehydrogenase (LDH) (U/L)	237	120-246
C-reactive protein (CRP) (mg/dL)	0.3	<1.0

**Figure 1:**
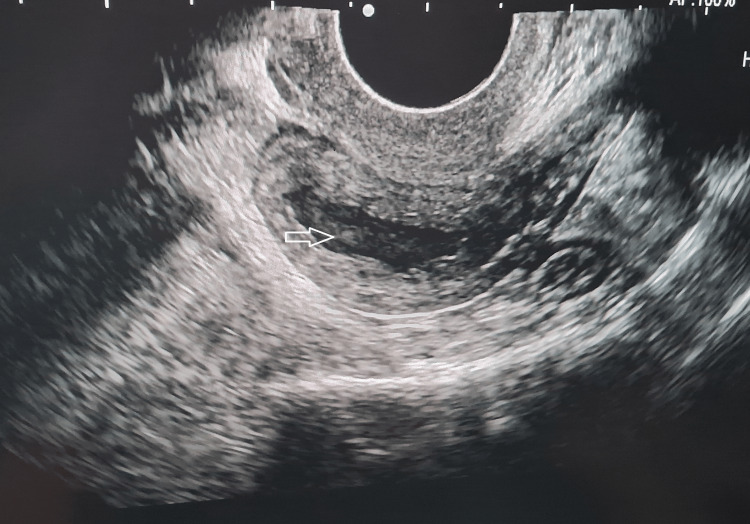
TVS image showing heterogeneous collection with multiple internal echoes within the endometrial cavity. TVS: trans-vaginal sonography

A transvaginal ultrasound was done which suggested an endometrial collection of size 3.3 x 3.1 x 2.2 cm with a volume of 12.7 cc in the endometrium. Densely packed internal echoes were observed within it, suggestive of pyometra (Figure 1).

The patient was started on intravenous meropenem, 1 gram three times a day, and tumour markers were sent to rule out malignancy. The culture report from the cervical swab showed the growth of *Pseudomonas aeruginosa* and the patient was continued with intravenous meropenem therapy for seven days as she was sensitive according to the culture.

**Figure 2:**
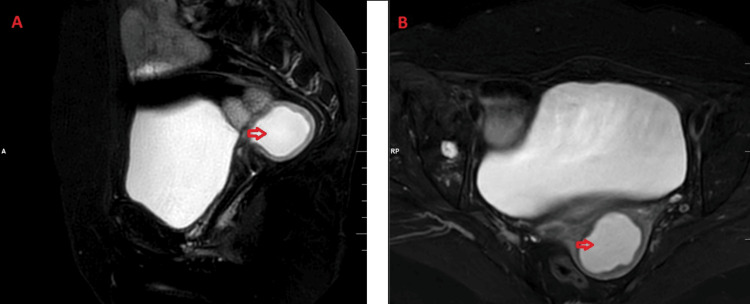
MRI T2-FS images of the pelvis (sagittal section: A, axial section: B) showing homogeneously hyperintense collection within the uterus. MRI: magnetic resonance imaging; FS: fat saturated

**Figure 3:**
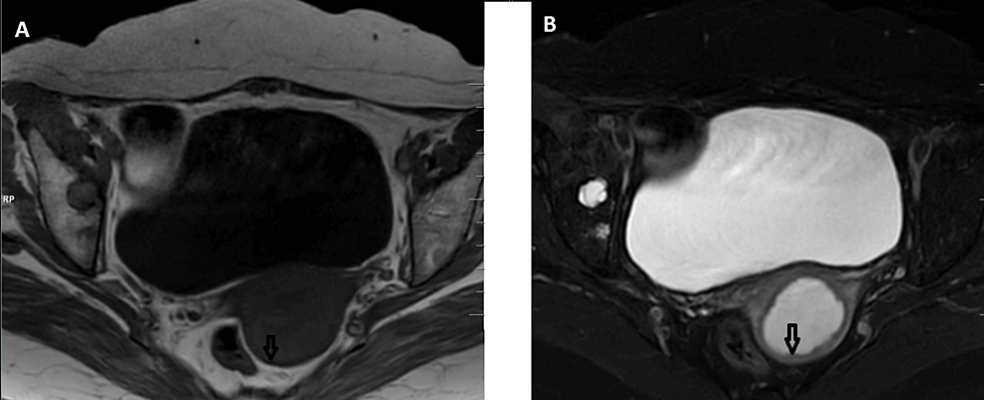
MRI pelvis T1 (A) and T2-FS (B) axial sections of the pelvis showing thinned-out endometrium. MRI: magnetic resonance imaging; FS: fat saturated

MRI of the abdomen and pelvis revealed an endometrium measuring 2.1 centimetres, showing homogeneous hyperintensity on T2-weighted images and hypointensity on T1-weighted sequences, with subtle restricted diffusion and a thinned endometrium on diffusion-weighted imaging (DWI) images, suggestive of pyometra (Figures 2, 3).

**Figure 4:**
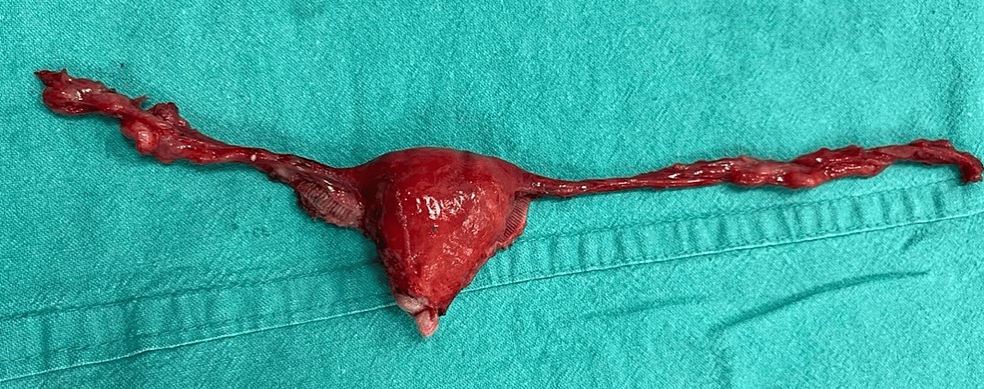
Small atrophied uterus with bilateral fallopian tubes

**Figure 5:**
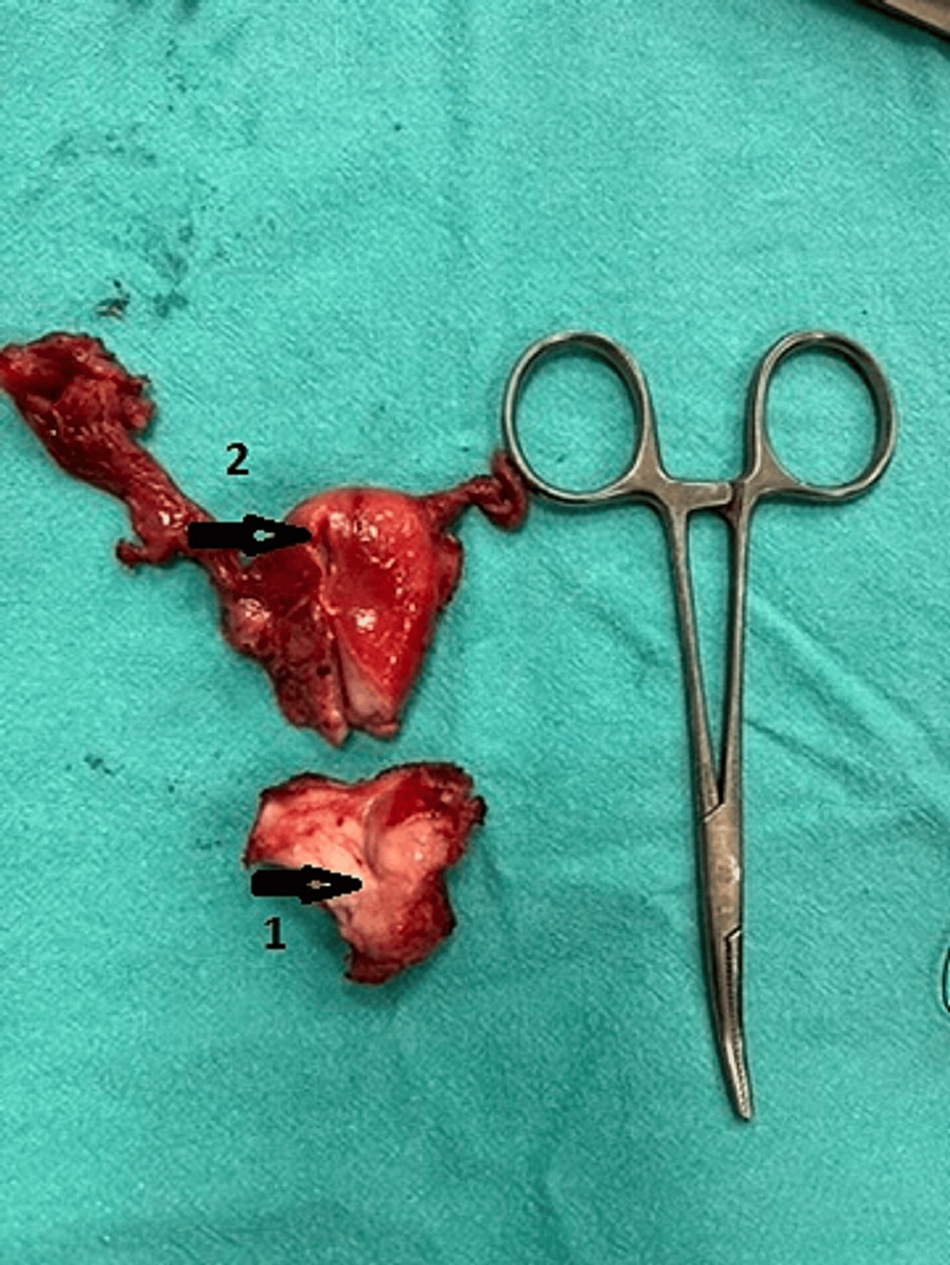
Small atrophied uterus (1) Atrophied cervical os, (2) thinned-out uterine walls.

When the patient was hemodynamically stable and the leucocyte count had come to normal values, the patient was posted for an elective hysterectomy to prevent the perforation of the uterus. Intra-op a small atrophic uterus measuring around 4x2.5x2 cm was removed along with bilateral fallopian tubes and ovaries (Figure 4). The uterus had thin walls and the cervix was hypertrophied (Figure 5). A frozen section of the uterus along with the cervix and fallopian tubes was sent to rule out malignancy, which was negative for the presence of any malignant lesion. The specimen was sent for histopathology. The procedure was uneventful. The patient has a steady recovery.

**Figure 6:**
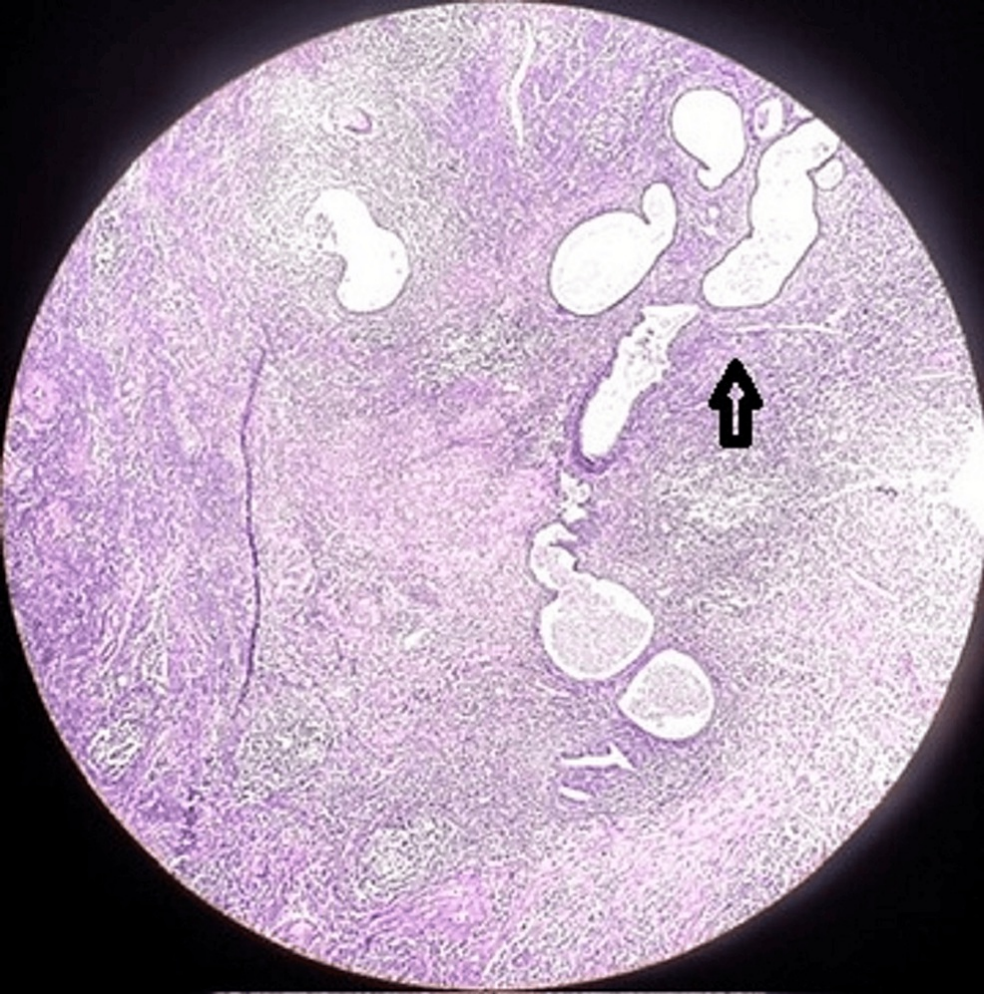
10X histopathological slide stained with haematoxylin and eosin showing cystic dilatation and atrophy of endometrial glands with infiltration of chronic inflammatory cells like lymphocytes and plasma cells.

The histopathology report was suggestive of endometrium showing senile cystic atrophy, chronic endometritis and hematopyometry. However, no evidence of adenocarcinoma of endometrium was seen on histopathology. The section from the cervix has features suggestive of chronic cervicitis with surface ulceration (Figure 6).

## Discussion

The accumulation of purulent fluid in the uterus is called a pyometra. Pyometra is reported to occur in 0.1-0.2% of all gynaecologic patients [8,9]. The internal os is most commonly affected by cervical stenosis. Cervical stenosis can be caused by neoplasia, radiation therapy, and iatrogenic events. These causes need to be treated promptly. Of these, idiopathic causes account for 74.1% of cases, genital tract anomalies for 3.7% of cases, and cancers for 22.2% of cases [10]. To exclude concomitant cancers, a thorough pelvic examination and history should be performed after a diagnosis. Pyometra is a critical medical disorder due to the risk of spontaneous perforation as well as its association with malignancies. Pyometra is a rare but serious gynecologic illness because spontaneous rupture can cause severe morbidity and mortality, and there is a substantial likelihood of connection with malignant disease.

Lower abdominal pain, postmenopausal bleeding, and purulent vaginal discharge are a common triad of symptoms associated with pyometra [4]. Under most circumstances, the preferred course of treatment is dilatation of the cervix, allowing pus drainage followed by hysteroscopic examination, which is avoided in this case as there were higher chances of uterine rupture. In these conditions, it is critical to distinguish between malignancy and exclude the potential of cancer. Past studies have demonstrated that cases that aren't associated with malignancy have a better prognosis than those that are [11,12]. In order to rule out cervical malignancy, the endometrial samples ought to undergo a thorough rigorous histological investigation [13,14]. It is recommended to culture the collected pus and perform both tubercular gene testing and sensitivity testing for specific antibiotic coverage. In certain cases, such as ours in which the uterine wall is thinned which may cause a perforation or in a case with sepsis and perforation, a hysterectomy may be necessary. When a patient is fit for surgery, a pan-hysterectomy should be performed within a month of receiving adequate drainage in cases of pyometra caused by senile endometritis, as recurrence is common in 2-11 months. Medical care with cyclic oestrogen therapy (Premarin 0.625 mg daily) for four to six months is effective for people who are not fit for surgery [15].

Mukai et al. drained their pyometra at three different intervals and gave intrauterine lavage [16]. Waghe et al. had done dilatation and curettage preceding with admission of higher antibiotics [1].

## Conclusions

Senile pyometra is a rare and grave condition which can lead to severe morbidity and mortality in the patient if left untreated. In cases like this, it is important to make sure that the uterus does not get perforated. Wherever necessary, a friable uterus biopsy can be postponed, preventing perforation. The pus drained should be sent for culture, and tuberculosis should always be ruled out. To measure and identify the type of fluid accumulated in the uterus and rule out uterine perforation, ultrasonography (USG) is the first and most crucial test. It is advisable to take into account CT scans and MRIs in order to quantify the collection and exclude pyoperitoneum.
